# Anti-quorum Sensing and Antimicrobial Effect of Mediterranean Plant Essential Oils Against Phytopathogenic Bacteria

**DOI:** 10.3389/fmicb.2019.02619

**Published:** 2019-11-19

**Authors:** Ippolito Camele, Hazem S. Elshafie, Lucia Caputo, Vincenzo De Feo

**Affiliations:** ^1^School of Agricultural, Forestry, Food and Environmental Sciences, University of Basilicata, Potenza, Italy; ^2^Department of Pharmacy, University of Salerno, Salerno, Italy

**Keywords:** natural products, postharvest disease, secondary metabolites, quorum sensing, biofilm

## Abstract

Essential oils (EOs) are one of the most interesting natural products extracted from different aromatic plants. For centuries, EOs have been considered an essential part of the traditional pharmacopeia. Many plant EOs have been reported as possible effective alternatives for commercial pesticides, and their single constituents have been used efficiently in food preservation for their promising anti-QS activity against several food pathogenic microorganisms. The current mini review gives a general overview over the microbicide effect as well as anti-quorum sensing and the anti-biofilm formation of some common plant EOs, especially those of *Lamiaceae* and *Verbanaceae* families; these are commonly grown in the Mediterranean region and are effective against some serious food phytopathogenic bacteria.

## Introduction

Essential oils (EOs) are considered important natural products extracted from aromatic plants and have been used for centuries in traditional pharmacopeia (Elshafie and Camele, 2017). EOs can be identified as concentrated hydrophobic liquids containing volatile aromatic compounds (Camele et al., 2012; Elshafie et al., 2015b). They have several biological, nutritional, and pharmaceutical properties. Historically, they represent an important part of the traditional pharmacopeia (Elshafie et al., 2015a). In addition, several EOs have demonstrated interesting antimicrobial effects against many serious phytopathogenic fungi and bacteria, both *in vitro* and *in vivo*, as well as an effective use in the production of pharmaceutical drugs for plant and human diseases (Mancini et al., 2014; Elshafie and Camele, 2017). Bacterial biofilm is considered a severe hygiene problem in the environment, plant and human health, and in the food industry. Biofilms make bacteria more resistant to disinfectants and different antimicrobial agents (Jamal et al., 2018). Many plant EOs have showed promising anti-biofilm formation and quorum sensing (QS) effects (Poli et al., 2018).

In this review, we give some more information about biofilm formation and the QS phenomenon, especially in food pathogenic bacteria. Moreover, this review illustrates the potential use of some plant EOs as anti-QS and biofilm agents to prevent bacterial infection and avoid the drug-resistance ability of many pathogenic bacteria.

## Biofilm Formation and Quorum Sensing

Biofilm formation is considered one of the most essential causes of bacterial resistance toward different traditional chemical and physical treatments and antimicrobial agents (Ivanova et al., 2018). Several animal and human microbial infections are related to microbial biofilm ability, which has recently become a real challenge (Coenye and Nelis, 2010).

Biofilm formation is highly related to the density-dependent cell communication called QS that plays an essential role in the biofilm development of many pathogenic microorganisms and triggers their resistance and virulence (Habeck, 2003). QS enables bacterial cells to have a multicellular behavior in prokaryotes and helps in regulating the virulence process, production of secondary metabolites, symbiosis, biofilm formation, induction of stationary phase responses, and motility for colony escape (Withers et al., 2001). QS allows bacteria cells to monitor their local population densities and regulate the timing of communal activities.

The most common bacterial food pathogens produce biofilms such as the *Pseudomonas* species, which are able to survive at high temperatures and reduce the shelf-life of foods and fish processing. In addition, *Bacillus cereus, Escherichia coli*, and *Staphylococcus aureus* were isolated from dairy processing lines, as reported by Kerekes et al. (2013).

Quorum sensing is a intercellular communication system that regulates microbe–microbe interactions (Nazzaro et al., 2019). The QS phenomenon regulates gene expression in response to the bacterial cell population size (Steindler and Venturi, 2007) and is expected to be the main function responsible for different bacterial phenotypes (Kumari et al., 2006; Duerkrop et al., 2007). Furthermore, most bacterial bioactive secondary metabolites are synthesized by stimulating some signal molecules that mediate the process of QS (Withers et al., 2001).

Several studies have shed light on the QS phenomenon in many gram-negative (G-ve) bacteria, including those pathogenic to plants and animals as well as human, such as the genus of *Agrobacterium*, *Aeromonas*, *Burkholderia*, *Chromobacterium*, *Citrobacter*, *Enterobacter*, *Erwinia, Hafnia*, *Nitrosomonas*, *Obesumbacterium*, *Pantoea*, *Pseudomonas*, *Rahnella, Ralstonia*, *Rhodobacter, Rhizobium*, *Serratia*, and *Yersinia.* In pathogenic species, the system may also enable coordination against the host, as in case of *Pseudomonas aeruginosa* infections in cystic fibrosis patients (Withers et al., 2001). Regarding *P. aeruginosa*, the opportunistic human pathogen, it secretes multiple extracellular virulence factors that cause extensive host tissue damage, and these factors are regulated by the QS phenomenon, as reported by Gera and Srivastava (2006).

## Signal Molecules Mediated Quorum Sensing

**FIGURE 1:**
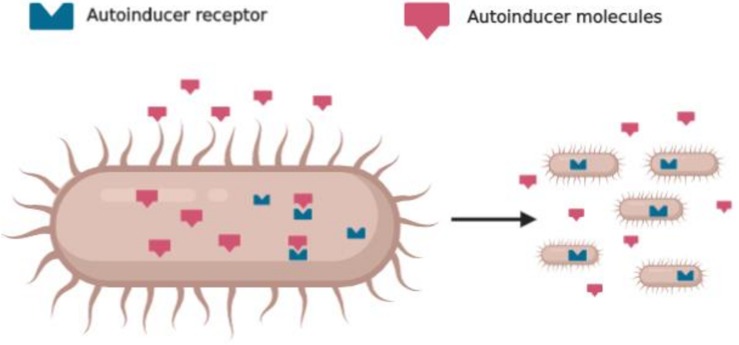
Schematic representation of quorum sensing: bacteria produce autoinducers molecules and detect the same molecules with specific receptors that coordinate their behavior.

The QS system is based on different key elements, such as autoinducers, signal synthase, autoinducers receptors, and regulated genes (Figure 1). In general, G-ve bacteria use the Lux-R/I-type and gram-positive (G + ve) bacteria use the peptide signaling system (Nazzaro et al., 2019). Signals molecules that mediate QS are oligopeptides in G + ve, *N*-Acyl-homoserine Lactones (*N*-AHLs) in G-ve, and a family of autoinducers known as autoinducer-2 (AI-2) in both G-ve and G + ve (Miller and Bassler, 2001). In particular, the formation and the activation of *N*-AHLs are directly proportional to the bacterial density that enables them to act as a multicellular organism and become ready to make behavioral decisions (Withers et al., 2001).

*N*-AHLs are considered the main player in the bacterial pathogenesis (Miller and Bassler, 2001) as well as disease suppression by certain plant beneficial bacteria (Zhou et al., 2003). When the production of *N*-AHLs reaches a specific threshold concentration, corresponding to a critical population density, these signals serve as co-inducers to regulate the transcription of bacterial target genes that are responsible for the pathogenicity and production of bioactive secondary metabolites. On the other hand, autoinducers can also regulate the transcription of some bacterial genes as much as the cell density (Smith et al., 2003). Autoinducers are able to coordinate a suite of virulence factors: antibiotic production (Stead et al., 1996), biofilm formation (Van Delden and Iglewski, 1998), luminescence, and swarming motility (Rasmussen et al., 2000).

## Anti-Biofilm and Quorum Sensing Effects of Eos

EOs are composed mainly from two groups of single substances, terpenoids (monoterpene, sesquiterpene and di-terpene) and phenylpropanoids (Nieto, 2017). The terpenoid group includes several compounds commonly present in the chemical composition of many plant EOs with different percentages, such as *p*-cymene, pinene, limonene, sabinene, and terpinene (Kerekes et al., 2015), geraniol, menthol, linalool, citronellol, carvone, thymol, carvacrol, geranyl acetate, eugenyl acetate, geranial, neral, and 1,8-cineole (Ayala-Zavala et al., 2007; Nieto, 2017). In addition, phenylpropanoids include several aromatic compounds such as cinnamyl alcohol, cinnamaldehyde, eugenol, and methyl cinnamate (Hyldgaard et al., 2012). Since not all of the above compounds have anti-QS activity, an overview of the principal constituents of some common plant EOs extracted from different aromatic plants and that display anti-QS activity are reported in Table 1.

**TABLE 1:** List of common plant EOs and their single constituents that display anti-QS activity.

**Plant scientific name**	**Family**	**Essential oils**	**Raw materials**	**Principal bioactive constituents**	**References**
*Carum carvi* (L.) Sprengel	*Apiaceae*	Caraway	Fruits	Limonene 51%, sabinene 0.3%, β-myrcene 0.4%, β-pinene 0.2%, linalool 0.2%, Bornyl acetate	Raal et al., 2012; Al-Haidari et al., 2016
*Origanum vulgare* L.	*Lamiaceae*	Oregano	Leaves	Carvacrol 75%, linalool 1.3%, limonene 1.3%, (E)-citral 2.5%, γ-terpinene 0.1%, 1,8-cineole 0.2%, eugenol 1.2%	Mancini et al., 2014; Elshafie et al., 2017; Asfour, 2018
*Majorana hortensis* L.	*Lamiaceae*	Marjoram	Flowers	α-pinene 9%, β-pinene 3.8%, limonene 6.4%, 1,8-cineole 33.5%, γ-terpinene 0.1%, linalol 9.8%	Kerekes et al., 2013; Elshafie et al., 2016a; Luciardi et al., 2016
*Thymus vulgaris* L.	*Lamiaceae*	Thyme	Leaves	Carvacrol 3.5%, *p*-cymene 11.2%, terpinene 4.8%	Elshafie et al., 2015a; Asfour, 2018
*Salvia officinalis* L.	*Lamiaceae*	Sage	Leaves	Camphor 13.9%, limonene 1.4%, α-pinene 4.4%, 1,8-cineole 4.2%	Elshafie et al., 2016a; Asfour, 2018
*Verbena officinalis* L.	*Verbenaceae*	Vervain	Leaves	Limonene 2.3%, 1.8-cineole, *cis*-Anethole 0.2%, linalol 0.1%, camphor 0.2%	Duke, 1992; Rehecho et al., 2011; Chalchat and Garry, 1995
*Lavandula stoechas* L.	*Lamiaceae*	Lavender	Flowers	Fenchone 34.9%, camphone 28.9%	Poli et al., 2018
*Citrus clementina* Hort. ex Tan.	*Rutaceae*	Clementina	Peel	Sabinene 31.4%, linalool 20.4%	Kerekes et al., 2013; Luciardi et al., 2016; Poli et al., 2018
*Murraya koenigii* (L.) Sprengel	*Rutaceae*	Curry tree	Leaves	Caryophyllene 9.49%, caryophyllene oxide 1.02%, α- and β-phellandrene 0.07%, α-Terpinene 2.37%, linalool 0.19%	Chowdhury et al., 2008; Bai and Vittal, 2014
*Hyptis suaveolens* L.	*Lamiaceae*	Pignut	Leaves	*Trans*-β-caryophyllene 11.3%, α-pinene (2.3), camphene 2.6%, β-myrcene 1.5%, *p*-cymene (11.2), limonene (7.2), γ-terpinene (1.5)	Stashenko et al., 2013
*Rosmarinus officinalis* L.	*Lamiaceae*	Rosemary	Leaves	α-pinene (26%), 1,8-cineole (25%), camphor 12%	Alvarez et al., 2012; Melito et al., 2019

In particular, the family *Lamiaceae* is considered one of the most important families of medicinal and aromatic plants; it includes *Origanum vulgare* L., *Majorana hortensis* L., *Thymus vulgaris* L., *Salvia officinalis* L., *Lavandula stoechas* L., *Hyptis suaveolens* L., and *Rosmarinus officinalis* L. Besides that, *Verbena officinalis* L. (*Verbenaceae*), *Carum carvi* L. (*Apiaceae*), *Citrus clementina* Hort. ex Tan. (*Rutaceae*), *Murraya koenigii* (L.), and Sprengel (*Rutaceae*) are also considered important aromatic plants where their single constituents have promising anti-QS properties to combat different food pathogenic microorganisms, as reported in Table 1.

*Origanum vulgare*, one of the most efficient plant EOs, is able to counteract biofilm formation and the QS mechanism with its main bioactive constituents (carvacrol), which has explicated a promising effect against different food and human pathogenic bacteria, such as *Salmonella enterica* subsp. *typhimurium* and *S. aureus* (Asfour, 2018). In addition, other single constituents of oregano EO, such as linalool, limonene, (E)-citral, γ-terpinene, 1,8-cineole, and eugenol, have exhibited anti-QS effects, as reported in several studies (Raal et al., 2012; Al-Haidari et al., 2016).

Moreover, clary sage, juniper, lemon, and marjoram EOs have been examined in the food industry and showed an effective anti-QS effect by preventing biofilm formation, especially against *B. cereus*, *E. coli*, and *Pichia anomala* (Kerekes et al., 2013; Luciardi et al., 2016).

Benzaid et al. (2019) studied the anti-biofilm formation effect of mint EO on *Candida albicans* and concluded that this EO has reduced the biofilm formation of *C. albicans*. Marjoram EO also showed a promising anti-QS effect against *Chromobacterium violaceum*, the positive sensor strains for AHL-mediated QS (Kerekes et al., 2013). Poli et al. (2018) reported that *Mentha suaveolens* ssp. *insularis* acts as an inhibitor of violacein production and the biofilm formation of *C. violaceum*, and the *Carum copticum* EO showed anti-QS activity against *C. violaceum* (Snoussi et al., 2018). Szabó et al. (2010) reported that EOs extracted from lavender, citrus, and rosemary plants can also inhibit QS and concluded that these EOs can be used in the pharmaceutical industry for discovering new therapy for serious human infections.

## Microbicide Effect of Plant Essential Oils

Many foodborn pathogenic bacteria (FBPB) produce serious toxins that lead to food spoilage and human infection. Some of FBPB are characterized by the abovementioned phenomena of QS, such as *E. coli*, *Listeria monocytogenes*, *Clostridium* spp., *S. enterica*, and *S*. aureus (Martinoviæ et al., 2016).

In general, the use of antibiotics are the most common substances for the direct controlling of whole bacteria (Poli et al., 2018); however its use in the food industry sector for controlling FBPB is prohibited in most developed countries to avoid creating different resistant human strains. Furthermore, many synthetic preservatives used in the food industry with antimicrobial effects may causing allergies, intoxications, cancer, and other degenerative diseases (Aminzare et al., 2016). For instance, the scientific research has continuously been carried out to present new substances that can be effectively used in controlling FBPB, particularly as biofilm preventers and for the inactivation of QS in the food industry, against physical, chemical, and/or natural substances such as nanoparticles, antimicrobial polymers, hydrogel, ozone, and extracellular hydrolytic enzymes (Elshafie and Camele, 2017; Jiang et al., 2019).

Recently, there has been a great revolution in scientific research regarding the importance of using plant EOs in combating many pathogenic bacteria, especially against food spoilage and for human health (Khan et al., 2009; Olivero et al., 2010; Camele et al., 2012; Nieto, 2017).

In particular, many interesting EOs and their single components, extracted from oregano, sage, marjoram, and vervain in particular, have been used effectively against some post-harvest diseases (Mancini et al., 2014; Elshafie et al., 2015a, 2016a, 2017). The three EOs extracted from *V. officinalis*, *M. hortensis*, and *S. officinalis* (Elshafie et al., 2016a) and those extracted from leaves and fruits of *Schinus terebinthifolius* (Elshafie et al., 2016b) showed promising antimicrobial activity against some serious phytopathogens such as *Colletotrichum acutatum*, *Botrytis cinerea*, *Clavibacter michiganensis*, *Xanthomonas campestris*, and *Pseudomonas syringae* pv. *phaseolicola*.

Other plant Eos, such as *O. vulgare*, *O. heracleoticum*, and *O. majorana*, showed effective microbicide effects against some post-harvest pathogenic fungi (*Aspergillus* sp., *Penicillium* sp., *Monilinia* sp., and *B. cinerea*) and some phytopathogenic bacteria (*Bacillus megaterium, C. michiganensis*, *X. campestris*, and *P. syringae* pv. *phaseolicola*) (Della Pepa et al., 2019).

## Mode of Action

Many researchers have hypothesized that the possible mechanism behind the bioactivity of many EOs is due to their principal bioactive single molecules. In particular, the use of single components to control biofilm formation could be, in some cases, sufficient, such as *B. cereus* and *E. coli* (Kerekes et al., 2013); however, other studies explained that the synergism between different single components display better effects, like in case of the *Listeria monocytogenes* biofilm, where the synergism between a-pinene, limonene, and linalool substances can be more effective than each single component (Sandasi et al., 2009).

In addition, the synergic effect between different single constituents could trigger the antimicrobial effectiveness of EOs and may reduce the resistance of many pathogenic microorganisms (Elshafie et al., 2015b). Some single constituents can damage the cell walls and plasma membranes of microbial cells, alter morphology, and increase cell permeability (Elshafie et al., 2019). Adebayo et al. (2012) reported that carvacrol, γ-terpinene, and *p*-cymene could be effective on their own and also have a synergic effect when they are combined. This synergistic effect is due to the action of *p*-cymene, which works as mediator for transportation of carvacrol and γ-terpinene across the cell wall and cytoplasmic membrane of pathogenic microorganisms. On the other hand, the lipophilic properties of many single components play a role in degrading the microbe plasma membrane and, thus, lead to the lyses of the hypha wall (Elshafie and Camele, 2017).

## Conclusion

The biofilm formation of pathogenic bacteria is considered a big challenge for the food industry and human/animal health. The QS mechanism regulates the bacterial biofilm formation; thus, destroying and/or disrupting this mechanism can help to prevent biofilm formation and then solve many health problems. Many plant EOs display promising anti-QS properties by preventing biofilm formation, which could be very important in reducing the virulence and pathogenicity of drug-resistant bacteria, especially for those that are food pathogenic. In fact, the use of plant EOs in food industry do not change the organoleptic properties of foods, and their use could thus be a promising natural alternative for several synthetic food preservatives. Finally, many plant EOs can represent a possible substitute for many traditional antimicrobial drugs, which have a significant negative impact on the environment and human/animal health.

## Author Contributions

IC and HE conceived the study, provided the table, and wrote the manuscript. LC and VD revised the manuscript and provided the figure.

## Conflict of Interest

The authors declare that the research was conducted in the absence of any commercial or financial relationships that could be construed as a potential conflict of interest.
